# Exercise intensity and cardiac metabolic adaptations in elderly heart failure rehabilitation: A review

**DOI:** 10.1016/j.ijcrp.2025.200525

**Published:** 2025-10-08

**Authors:** Morteza Esmaeili, Riyas Vettukattil

**Affiliations:** a Department of Electrical Engineering and Computer Science, Faculty of Science and Technology, University of Stavanger, Stavanger, Norway; b Faculty of Medicine, Institute of Clinical Medicine, University of Oslo, Oslo, Norway; c Division of Pediatric and Adolescent Medicine, Oslo University Hospital, Oslo, Norway

**Keywords:** Cardioprotection, Cardiac metabolism, Metabolic remodeling, Mitochondrial function, High-intensity exercise, Moderate intensity exercise

## Abstract

**Introduction:**

Heart function depends on efficient cardiac metabolism and energy production. Exercise is vital for supporting these processes, especially in heart failure (HF) rehabilitation, where weakened metabolism and energy deficits are common. Structured exercise improves metabolic efficiency while reducing sedentary behavior, such as prolonged sitting, further supports heart health. Even in regular exercisers, prolonged sitting increases cardiovascular risks.

**Method:**

This review analyzed studies from 2017 to November 2024 on how exercise intensity affects cardiac metabolism, energy production, and mitochondrial function in HF patients. Searches were conducted in PubMed, Scopus, and Google Scholar using terms like "exercise intensity" and "cardiac metabolism." Studies focused on metabolic changes and mitochondrial bioenergetics were included; those lacking relevant data were excluded.

**Results:**

Structured exercise combined with reduced sedentary time significantly improves heart health in HF patients. Exercise gradually strengthens heart muscle, enhances energy utilization, and improves the heart's ability to handle daily stress. Reducing sedentary behavior complements these effects by lowering cardiovascular risks.

**Conclusion:**

Improving heart health in HF patients requires both regular exercise and minimized sedentary time. Physical activities can enhance cardiac metabolism, energy efficiency, and overall heart function, providing an effective pathway for better outcomes.

## Highlights

• Exercise-Based Rehab is a Class I recommendation for heart failure(HF), improving exercise capacity & quality of life.
• High-Intensity Interval Training (HIIT) improve exercise capacity and reduces symptom burden in HF patients.
• Exercises like resistance, inspiratory HIIT, and remote ischemic conditioning may improve function in HF patients.
• Regular exercise is crucial, but prolonged sitting cardiovascular risk factor.

## 1 Introduction

Sustaining heart functions rely on proper cardiac metabolism and efficient energy production. Exercise physiology plays a pivotal role in enhancing cardiac metabolism, which is relevant for the rehabilitation of heart failure (HF), a condition that is characterized by impaired cardiac metabolism and bioenergetic deficits [1,2]. Exercise can improve metabolic efficiency and facilitate substantial adaptations in cardiac muscle tissue, impacting the remodeling processes and the physiological stress responses of the myocardium [3,4] (see Table 1).

**Table 1:** Cardioprotective mechanisms and exercise.

Cardioprotective protein/mechanism	Exercise types	Modulation mechanism	Cardioprotection effects	Key findings
Follistatin-like 1 (FSTL1) [42]	Intense aerobic	Increases FSTL1 in cardiac/skeletal muscles.	Promotes blood vessel growth and reduces fibrosis.	Supports heart repair after myocardial infarction.
Natriuretic peptides (ANP, BNP) [4,45]	Aerobic and resistance	Released from the heart in response to stretch and changes in blood volume.	Improves vascular function and helps control blood pressure.	Structured exercise regulates ANP/BNP and reduces N-terminal pro-B-type natriuretic peptide (NT-proBNP) levels for long-term heart protection.
Fibroblast growth factor 21 (FGF21) [46]	High-intensity interval training (HIIT)	Released from liver/adipose tissue during exercise.	Enhances heart metabolism and reduces hypertrophy.	HIIT boosts FGF21, improving heart health
Endothelial nitric oxide synthase [26]	Aerobic exercise	Improves vasodilation and blood flow to the heart.	Exercise upregulates eNOS, supporting long-term heart health.	Improves vasodilation and blood flow to the heart.
Ischemic preconditioning [26]	Aerobic (pre-ischemic exercise)	Mimics short-term ischemia to reduce future damage.	Boosts heart resilience and minimizes injury.	Pre-ischemic exercise enhances heart protection against ischemia.
Sirtuins (SIRT1, SIRT3) [47,48]	Aerobic & Resistance	Exercise-induced upregulation of SIRT1	Improves mitochondrial function and reduces oxidative stress.	Exercise upregulates SIRT1, enhancing mitochondrial function and reducing oxidative stress.
Exosomes and MicroRNAs [39,44]	Various types of exercise	Exercise-induced exosomes carry cardioprotective microRNAs.	Supports metabolic adaptation and mitochondrial health.	Exercise-induced microRNAs promote heart protection, improve endothelial function, reduce inflammation, and enhance antioxidant defenses.
Myokines – Brain-derived neurotrophic factor (BDNF) [22,36]	Aerobic (high-intensity)	Boosts BDNF levels via muscle-brain interaction.	Lowers cardiovascular risk and supports heart plasticity.	High-intensity exercise improves mitochondrial metabolism, supports cardiac bioenergetics, and improves fatty acid oxidation.
Myokines – Irisin [49]	Resistance and combined exercise	Released during muscle contraction	Improves fat oxidation, and glucose metabolism, and reduces inflammation.	Irisin triggers antioxidant defenses and activates heart-protective pathways, improving mitochondrial metabolism.
Exercise-Based Cardiac Rehabilitation (ExCR) [24]	Various types of exercise	Enhances heart function and improves outcomes in heart failure.	ExCR enhances heart function and the quality of life in patients.	ExCR improves functional capacity and reduces oxidative stress.
Remote ischemic conditioning (RIC) [40,50]	Various types of exercise	RIC induces ischemia in limbs to protect the heart.	RIC reduces infarct size, improves outcomes, and enhances left ventricular ejection fraction.	RIC shows promise in reducing heart attack damage.

Recent research emphasizes the importance of reducing sedentary behaviors, like prolonged sitting, to improve cardiovascular health [5,6]. These studies show that sitting, even with regular exercise, increases cardiovascular risks. Increased daily movement, such as standing, walking, or light physical activity, offers significant benefits to high-risk individuals or those with chronic conditions [7]. This highlights the need for both structured exercise and reduced sitting time to enhance cardiovascular health. This review aims to elucidate the novel impact of exercise intensity on cardiac metabolism and myocardial bioenergetics, specifically highlighting crucial metabolic alterations and protective effects of exercise training in elderly heart failure patients, an area underrepresented in current literature. Our investigation highlights the novel effects of exercise intensity on cardiac metabolism and myocardial bioenergetics, specifically in elderly heart failure patients, an area that has been largely underexplored in existing literature.

## 2 Method

### 2.1 Search strategy and data sources

Inclusion criteria were relevant studies published between 2017 and November 2024. A comprehensive search was conducted in three electronic databases, PubMed, Scopus, and Google Scholar, using relevant keywords and controlled vocabulary terms, such as “exercise intensity,” “cardiac metabolism,” “mitochondrial function,” “elderly,” and “heart failure”, combined using Boolean operators (AND/OR). In (i) Scopus: ((exercise intensity) AND (Elderly) AND (cardiac metabolism) AND (mitochondrial function) AND (heart failure)) AND PUBYEAR >2016 AND PUBYEAR <2025 AND (LIMIT-TO (SUBJAREA, "MEDI")) AND (LIMIT-TO (DOCTYPE, "ar")) AND (LIMIT-TO (LANGUAGE, "English")); (ii) PubMed ((Exercise intensity) AND (Elderly) AND ((Cardiac metabolism) OR (Mitochondrial function)) AND (Heart failure)); and in (iii) Google scholar: *Advanced search with all of the word*: “Elderly” "mitochondrial function" “cardiac metabolism” "heart failure" & *with the exact phrase of* "exercise intensity".

**Fig. 1:**
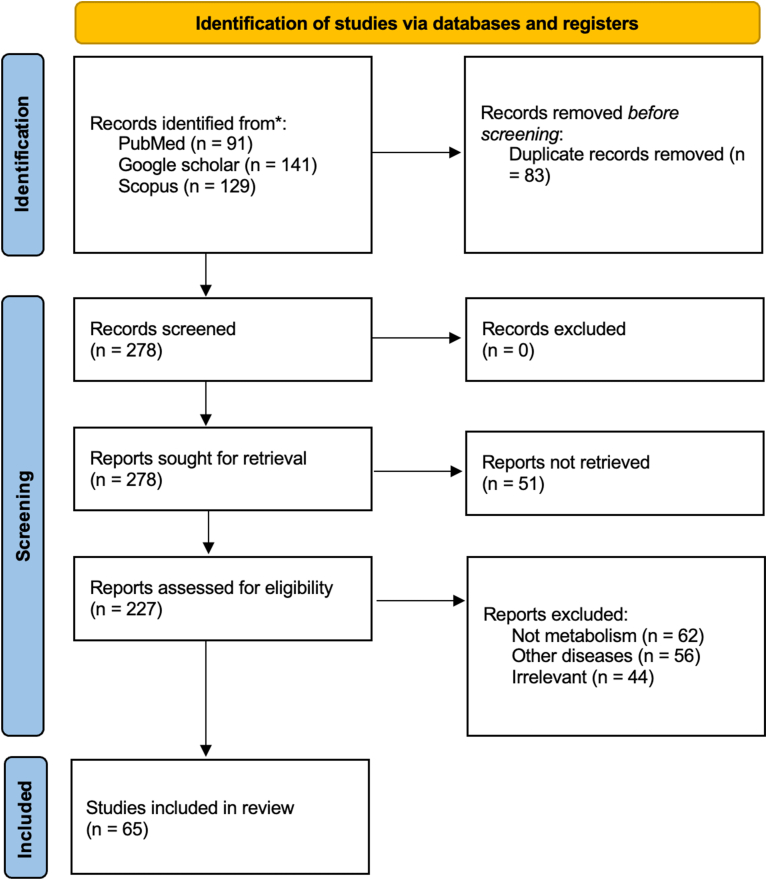
PRISMA flowchart of study selection.

After initial retrieval, duplicates were removed, and the remaining articles were screened by title and abstract. Full texts of eligible articles were reviewed for inclusion. A total of 361 articles were identified, with 65 meeting the final inclusion criteria. The article selection process is illustrated in the PRISMA flow diagram (Fig. 1). Studies were included if they explicitly examined the effects of varying exercise intensities on cardiac metabolism, with particular emphasis on mitochondrial bioenergetics and cardiac performance. Exclusion criteria comprised studies that failed to provide empirical data regarding the metabolic changes induced by differing exercise intensities or lacked rigorous experimental frameworks.

### 2.2 Eligibility criteria

To be included in the review, studies had to examine how different intensities of exercise impacted cardiac metabolism, energy production, and mitochondrial bioenergetics in individuals with HF. Only peer-reviewed studies that provided specific experimental data on metabolic changes and mitochondrial function were included. Studies that did not report relevant data, such as those lacking a focus on exercise intensity or lacking information on metabolic and mitochondrial outcomes, were excluded. The inclusion criteria were (i) Studies published between 2017 and November 2024; (ii) Research focused on HF patients (both chronic and acute); (iii) Studies examining exercise intensity and its impact on cardiac metabolism, mitochondrial function, and bioenergetics; and (iv) Peer-reviewed studies published in English.

The exclusion criteria were (i) Studies not involving exercise interventions or those without a focus on exercise intensity; (ii) Research that did not measure or report data on metabolic changes, mitochondrial function, or cardiac bioenergetics; (iii) Studies conducted on populations without HF (e.g., healthy individuals or those with other cardiovascular conditions); and (iv) Non-peer-reviewed articles (e.g., abstracts, reviews, or editorials).

To ensure reliable and rigorous findings, we assessed study quality using JBI Critical Appraisal Tools tailored to each study design. These tools offered criteria to assess methodology, bias, and evidence credibility. Based on this appraisal, each study was assigned a quality rating of "high," "moderate," or "low." This step supported interpretation, evidence-weighted conclusions, and transparent, evidence-based decision-making.

### 2.3 Study selection process

The study selection process was conducted in a systematic manner and encompassed three key stages.

a) **Initial Screening:** Two independent reviewers initially screened the titles and abstracts of identified articles to exclude those that evidently did not align with the inclusion criteria.
b) **Full-Text Assessment:** Following the initial screening, full-text articles were reviewed for adherence to the eligibility criteria. This assessment involved a detailed analysis of how each study addressed the specific research questions concerning the effects of exercise intensity on cardiac metabolism.
c) **Consensus Resolution:** In cases of disagreement regarding the eligibility of specific studies, reviewers engaged in thorough discussions to reach a consensus.

### 2.4 Data extraction

Data extraction was performed by two independent reviewers who independently recorded relevant information, including study design, sample characteristics, exercise protocols, outcomes related to cardiac metabolism, mitochondrial function, and exercise intensity levels. Any discrepancies between reviewers were resolved through discussion, and consensus was reached. The extracted data were systematically organized for analysis.

## 3 Results

### 3.1 Cardiac metabolism in heart failure

Cardiac function depends on efficient energy production, primarily through adenosine triphosphate (ATP) synthesized in mitochondria. Key mitochondrial complexes, such as Complex I and Complex II, play crucial roles in driving ATP production to meet the heart's energy demands. Impairments in mitochondrial function, often seen in HF, lead to energy deficits that exacerbate cardiac dysfunction [8]. Heart failure is marked by reduced phosphocreatine (PCr)/ATP ratio, altered glucose metabolism, and impaired mitochondrial function, limiting the heart's ability to adapt to stress [1]. Therapeutic interventions aim to restore metabolic balance, particularly by addressing disturbed creatine metabolism, a crucial factor for maintaining ATP levels during energy turnover. Resistance training has shown promise in improving creatine metabolism and energy efficiency [1,9].

Mitochondrial dysfunction in HF leads to a shift from fatty acid to glucose utilization, a less efficient energy source [8]. Physical trainings reverse this trend by enhancing fatty acid oxidation and increasing mitochondrial enzyme activity, improving energy production efficiency [10]. Exercise also facilitates systemic coordination of energy, optimizing the transport of glucose, lactate, and fatty acids between organs. Exercise improves mitochondrial efficiency and biogenesis by activating peroxisome proliferator-activated receptor gamma coactivator 1α (PGC-1α), which stimulates new mitochondria formation [11]. These adaptations enhance energy capacity, reduce oxidative stress, and improve cardiac function in HF [12,13].

Recent studies have highlighted the involvement of specific amino acids, particularly those linked to energy metabolism, such as branched-chain amino acids (BCAAs), including leucine, isoleucine, and valine, in the pathogenesis of HF [14]. Failing hearts show reduced BCAAs catabolism and accumulation of toxic metabolites. Under stress, such as during pressure overload or hypoxia, the heart may initially upregulate oxidation of BCAAs as a compensatory mechanism. But prolonged reliance on this pathway leads to maladaptation, worsening energy inefficiency and myocardial injury. Alterations in BCAAs metabolism can impair mitochondrial respiration, enhance oxidative stress, and contribute to cardiac dysfunction [14,15], and have been proposed as potential biomarkers reflecting altered metabolic states associated with cardiac dysfunction [14,16,17]. Including such biomarkers could enhance early detection and monitoring of HF progression.

**Fig. 2:**
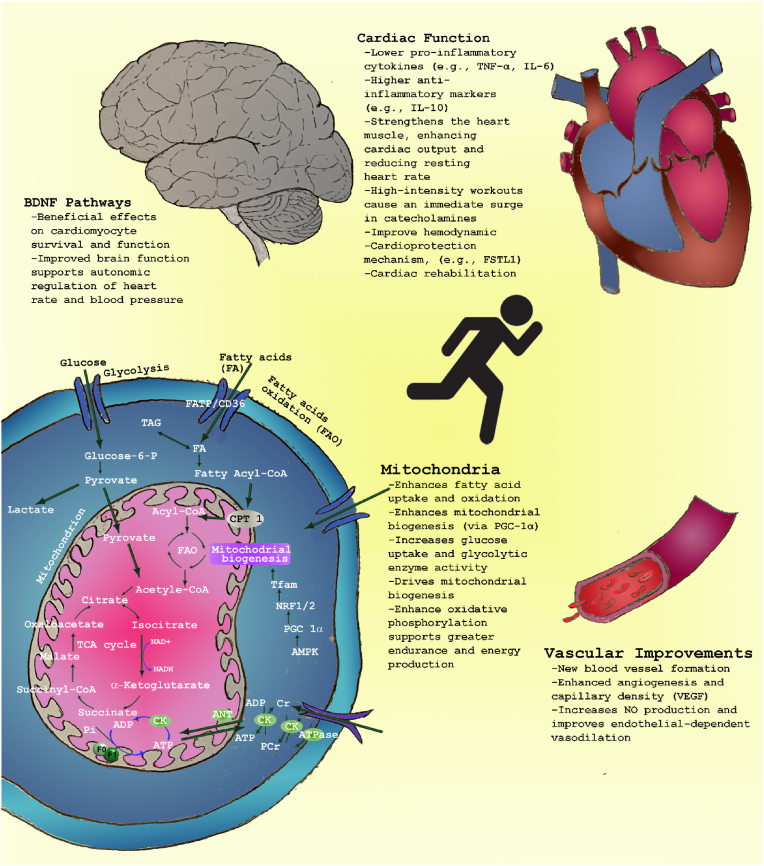
**Exercise-induced biological impact on the brain and cardiovascular system.** This schematic illustrates how physical activity stimulates molecular and cellular pathways that enhance cardiovascular and neurological health. In the cardiovascular system, exercise increases nitric oxide (NO) production, reduces pro-inflammatory cytokines such as tumor necrosis factor (TNF), and decreases reactive oxygen species (ROS). It also enhances fatty acid oxidation (FAO) through transporters like CD36/FATP and enzymes such as AMPK and CPT1. In the brain, exercise upregulates neurotrophic factors like brain-derived neurotrophic factor (BDNF) and promotes mitochondrial biogenesis via PGC-1α and Tfam. These effects contribute to improved energy metabolism (ADP/ATP, PCr, Cr, Pi) and angiogenesis via vascular endothelial growth factor (VEGF). Anti-inflammatory cytokines such as IL-6 and IL-10 are also involved. Abbreviations: CD36/FATP transporters; AMPK-activated protein kinase; PGC-1α, peroxisome proliferator-activated receptor gamma coactivator 1α; CPT1, carnitine palmitoyltransferase 1; ADP, adenosine diphosphate; ATP, adenosine triphosphate; PCr, phosphocreatine; Cr, creatine; Pi, inorganic phosphate; FSTL1, follistatin-like 1; IL-6, interleukin-6; IL-10, interleukin-6; Tfam, mitochondrial transcription factor A; VEGF, vascular endothelial growth factor; TCA, tricarboxylic acid cycle.

The systemic effects of exercise on the cardiovascular system are illustrated in Fig. 2, highlighting key molecular pathways such as nitric oxide production, mitochondrial biogenesis, and anti-inflammatory signaling. In summary, cardiac metabolism in heart failure is characterized by profound shifts in substrate utilization, reduced mitochondrial function, and an overall energy deficit. Understanding these metabolic alterations provides critical insights into the pathophysiology of HF and opens potential therapeutic pathways to ameliorate energy deficiency and improve cardiac function.

### 3.2 Exercise intensity and cardiac adaptation

Exercise, such as endurance and interval training, affects cardiovascular health differently. Endurance training improves aerobic capacity and cardiac efficiency, while high-intensity interval training (HIIT) enhances mitochondrial function and VO_2max_ (maximal oxygen uptake, a key fitness indicator) [18,19]. Both moderate and high-intensity exercise training improve VO_2max_ [19,20]. These exercises restore mitochondrial respiration, improve energy metabolism in the heart, and support cardiac health, particularly during stress conditions such as heart failure [21]. Endurance and interval training recruit different muscle fibers and metabolic pathways [22]. Endurance training targets slow-twitch fibers, increasing mitochondrial density, efficiency, and stroke volume, while HIIT engages both slow and fast-twitch fibers, improving mitochondrial function, VO_2max_, and metabolic flexibility [7,23,24].

Both endurance and high-intensity exercises improve mitochondrial biogenesis, with HIIT inducing a greater response [18]. Mitochondrial biogenesis occurs when the AMPK/PGC-1α signaling pathway is activated [12]. During high-intensity exercise, this pathway detects low energy and begins creating new mitochondria. HIIT and sprint interval training induce greater mitochondrial biogenesis and VO_2max_ changes than moderate continuous training [7,19]. These new mitochondria increase oxidative capacity and enhance mitophagy, improving mitochondrial health and efficiency. Sprint interval training improves rapid muscle glycogen depletion and activation of energy-sensing pathways, despite lower training volume [23]. Exercise intensity plays a much larger role than volume in mitochondrial adaptation.

In addition to metabolic adaptations, high-intensity exercise enhances endothelial-dependent vasodilation through the increased production of nitric oxide, this mechanism augments oxygen delivery to both the active skeletal muscles and the myocardium, thereby optimizing cardiovascular efficiency and reducing overall cardiac workload and stress [[25], [26], [27]]. Conversely, while physical training is documented to improve VO_2max_ and mitochondrial function, certain biomarkers, such as the PCr/ATP ratio, may not exhibit significant changes. This discrepancy occurs even in the presence of enhanced cardiac performance, thus indicating limitations in these markers for assessing the full spectrum of exercise-induced cardiovascular adaptations [19].

Recent studies have also highlighted the modifying effect of cardiorespiratory fitness on the relationship between obesity and HF outcomes [28,29]. These findings suggest that, despite the association between obesity and increased HF risk, intensive exercise training can significantly improve prognosis, supporting the role of structured exercise interventions in this population. Investigations on non-pharmacological therapies in HF, the authors highlight obesity as a significant risk factor, contributing directly via myocardial fat accumulation and indirectly through related comorbidities like hypertension and diabetes, which activate detrimental pathways such as RAAS and lead to cardiac fibrosis [29]. Although intentional weight loss reduces the HF incidence, the so-called *obesity paradox* suggests that lower BMI may paradoxically worsen outcomes in patients with established HF, especially when weight loss is unintentional [29].

### 3.3 High-intensity training derives distinct metabolic shifts and adaptation

Exercise triggers heart adaptations through metabolic and molecular pathways that support energy production and remodeling. Physiological remodeling occurs with high-intensity exercise in healthy individuals, strengthening heart muscles, improving efficiency, and boosting mitochondrial function. Pathological remodeling, linked to underlying conditions or excessive exercise, causes scarring and reduced efficiency. Exercise intensity also affects substrate utilization, with moderate exercise increasing fatty acid oxidation and high-intensity exercise promoting glucose use [7,25].

Aging and genetic factors also play a significant role in how individuals respond to exercise. Studies [[30], [31], [32]] suggest that intrinsic aerobic capacity and aging interact with exercise intensity to shape metabolic adaptations. Aging impacts metabolic adaptations to exercise primarily through a decline in mitochondrial function, reduced muscle mass (sarcopenia), and decreased cardiovascular efficiency [23,31,32]. Older individuals experience less effective mitochondrial biogenesis and lower improvements in VO_2max_ compared to younger populations. Aging also reduces metabolic flexibility, slowing the ability to switch between fuel sources during exercise [23,33]. Additionally, chronic inflammation and hormonal changes impair muscle recovery and adaptation, leading to slower and smaller gains from high-intensity exercise [27,34]. Despite these challenges, regular exercise can still offset age-related declines and improve overall health.

Several signaling pathways drive mitochondrial adaptations, including the AMPK and PGC-1α [12,25]. These pathways are involved in regulating mitochondrial biogenesis, fatty acid oxidation, and responsive energy production to account for increased physical activity [25]. PGC-1α expression increases significantly during exercise [11], which in turn promotes enhanced mitochondrial function and ATP production. This process is required to support elevated energy demands during prolonged or high-intensity exercise, ensuring a sustained elevated cardiac workload. The AMPK pathway acts as a cellular energy sensor. During exercise, when ATP levels drop, AMPK becomes activated, initiating a series of metabolic responses that ensure a continuous energy supply to the heart. Specifically, AMPK enhances glucose uptake and fatty acid oxidation, key processes for maintaining energy balance. Beyond this, the AMPK-PGC1α pathway plays a role in supporting overall mitochondrial health, helping the heart sustain its function during extended periods of physical activity. Studies examining myokines such as irisin and Brain-Derived Neurotrophic Factor (BDNF) on heart failure have demonstrated that the beneficial effects of myokines are mediated through the activation of the AMPKα-PGC1α signaling pathway [35,36].

Exercise induces physiological cardiac hypertrophy, distinct from pathological hypertrophy linked to conditions like hypertension. Mediated by IGF-1 (Insulin-like Growth Factor 1) and Akt signaling, exercise-induced hypertrophy promotes healthy cardiac cell growth, enhancing myocardial performance and cardiac output while supporting cardiovascular health. Exercise also helps regulate blood pressure, especially in hypertensive individuals. Moderate to high-intensity workouts, including HIIT, significantly lower blood pressure short- and long-term [37]. This post-exercise hypotension effect is more pronounced and sustained with higher-intensity activities, offering substantial benefits for hypertension management [37].

Mitochondrial efficiency is another notable outcome of regular exercise. Exercise enhances the oxidative capacity of mitochondria by improving the function of electron transport chain complexes Complex I and Complex II. This improves oxygen utilization and more efficient energy production, which is critical for endurance-based and high-intensity forms of exercise. By optimizing mitochondrial function, exercise improves the immediate performance of the heart and enhances its long-term resilience, especially for patients with HF [18,19].

One of the most immediate changes during HIIT is the rapid increase in lactate production [10]. Since our body is demanding energy at a rate faster than its capacity to supply through aerobic metabolism, it shifts to anaerobic pathways, thus producing lactate as the byproduct of metabolic breakdown [10]. This initial buildup can lead to the familiar muscle burn and eventually fatigue. Over time, however, the body adapts to this stress. Consistent high-intensity training can improve muscle performance in exchanging lactate in the bloodstream efficiently [10,18,19]. This pushes the lactate threshold higher, enabling sustained higher intensities of exercise longer without fatigue. It is an extremely important adaptation to endurance and performance in highly intense bouts of exercise.

Another key element of the metabolic adaptations associated with HIIT is the profound endocrine response [21]. High-intensity workouts immediately raise catecholamines, which include such energizing hormones as adrenaline and noradrenaline [26,38]. These resources then break down more fats, and after that process, more energy will become available for muscles. Immediately after exercise, hormones such as growth hormone and testosterone—which surged post-workout—play critical roles in muscle recovery and development [21,39]. These hormonal changes signal that the body will be able to meet the demands of HIIT and can also build a more resilient system against those demands in the future.

The human body gradually adapts to high-intensity exercise by increasing capillary density through angiogenesis, improving oxygen delivery and metabolic efficiency [10,23]. This enhances muscle endurance by better-handling oxygen delivery and waste removal, allowing for more effective recovery between high-intensity efforts [11,25].

HIIT activates the sympathetic nervous system, increasing heart rate, blood pressure, and blood flow to muscles [25,40]. Over time, the body becomes more efficient at managing these responses, improving cardiovascular performance both during exercise and at rest. Muscles also become better at absorbing glucose, enhancing insulin sensitivity, and reducing the risk of insulin resistance, particularly in those predisposed to metabolic conditions like Type 2 diabetes [1,7,33,40]. Regular high-intensity exercise promotes better blood sugar regulation and metabolic health.

Energy requirements of high-intensity exercise are not finished when the workout is finished. Following an aggressive workout, the body undergoes excess post-exercise oxygen consumption (EPOC) [41]. It is the heightened calorie burn that continues long after the body is restoring homeostasis. This phase includes repairing mechanisms for muscles, re-filling energy stores, and clearing metabolic byproducts. The more intense the exercise, the greater and longer the EPOC effect and the longer the post-workout calorie burn [41]. High-intensity exercise targets the fast-twitch muscle fibers (Type II), which are designed for power and speed, and tire much faster. HIIT increases the efficiency and endurance of the fast-twitch fibers, enabling them for longer-term, repeated bouts of high-intensity effort [13,34]. This is why increased speed and explosive strength often occur together for athletes who regularly train at high intensities.

### 3.4 Exercise-induced cardioprotection

Cardioprotection mechanisms include enhancing cardiac function, reducing oxidative stress, and promoting mitochondrial repair. Follistatin-like 1 (FSTL1) activity is involved in several cardioprotection physiological mechanisms [42]. Intense aerobic exercise increases FSTL1 expression in cardiac and skeletal muscles [42], promoting angiogenesis, reducing fibrosis, and activating TGFβ-Smad2/3 signaling for cardiac repair. This supports new blood vessel formation while limiting collagen deposition that impairs function. Exogenous FSTL1 may mimic exercise-induced cardioprotective effects, highlighting its therapeutic potential in post-MI recovery [42].

For individuals suffering from HF, especially those with a reduced ejection fraction, HIIT may or may not be useful in the context of HF rehabilitation [18]. For those patients’ cohorts proven to be useful, HIIT can boost VO_2peak_ (a measure of peak oxygen uptake) by as much as 20 %, while also decreasing the size of the left ventricle during contraction (left ventricular end-systolic diameter). These changes improve how well the heart pumps blood. This enhanced mitochondrial efficiency helps patients tolerate exercise better and has longer-term benefits, with research indicating that HIIT can reduce mortality rates over five years by around 60 % in HF patients [18].

Regular exercise acts as ischemic preconditioning (IPC), training the heart to resist ischemia by triggering protective mechanisms during short ischemia-reperfusion episodes. Exercise mimics IPC, introducing mild, controlled stress that enhances stress resistance [26]. It activates molecular pathways, increasing nitric oxide production and anti-radical defenses, reducing oxidative stress, and preventing cardiovascular damage. Additionally, exercise-induced activation of ATP-dependent potassium channels helps protect against chemotherapy-induced cardiotoxicity and long-term stressors.

Medications such as beta-blockers, angiotensin receptor blockers, sacubitril/valsartan, and ivabradine enhance HF management by improving diastolic function and exercise capacity. These drugs allow better chamber filling during diastole, with ivabradine and sacubitril/valsartan showing benefits in diastolic function and exercise performance [43]. Meta-analyses report significant gains in VO_2peak_ and 6-min walk test outcomes, though these improvements do not consistently reduce hospital readmissions or mortality. Still, their positive impact on exercise tolerance makes them valuable complements to exercise regimens, improving the quality of life in HF patients.

Exercise induces the release of exosomes containing irisin, a muscle-derived hormone with anti-inflammatory and antioxidant properties [13]. Irisin and specific microRNAs contained within these exosomes activate protective cardiovascular networks [13]. By supporting mitochondrial health and metabolic adaptation through pathways such as AMPK/PI3K/AKT and TGFβ1/Smad2/3, irisin contributes to long-term heart health and resilience to stress. This emerging area of research illustrates how exercise-mediated molecular signaling extends beyond immediate metabolic changes, promoting systemic cardiovascular protection. For example, previous studies showed that exercise upregulates the expression of miR-126 in endothelial progenitor cell-derived exosomes, which is known to inhibit the TGF-β/Smad3 signaling pathway, which is involved in cardiac fibrosis [44].

### 3.5 Exercise training and rehabilitation in heart failure

Exercise-based cardiac rehabilitation (ExCR) can improve exercise capacity and health-related quality of life in patients with HF [24]. Improvements in a 6-min walk test and reductions in scores on the Minnesota Living with Heart Failure Questionnaire demonstrate the effectiveness of ExCR as a Class I recommendation for HF management [24]. Importantly, these benefits were seen across diverse patient populations, irrespective of age, gender, or ejection fraction, suggesting that ExCR is broadly applicable and beneficial for patients with HF. Also, ExCR enhances SIRT1 activity in patients with HF, leading to increased levels of β-hydroxybutyrate, a ketone body that promotes metabolic efficiency and reduces oxidative stress [47]. SIRT1 is a protein that enhances mitochondrial biogenesis, improves metabolic efficiency, and reduces oxidative stress. As outlined in several studies [48], regular physical exercise upregulates SIRT1 expression, which is closely tied to enhanced mitochondrial function and resilience to metabolic and age-related diseases [38]. SIRT1 deacetylates and activates transcriptional coactivators like PGC-1α, a key regulator of fatty acid oxidation and mitochondrial efficiency. SIRT1 enables the heart to efficiently maintain energy production during excess physical activities, exercise, and fasting [51]. This cardiac adaptation reduces the risk of ischemic damage and other cardiovascular complications [51].

Exercise training is crucial in HF rehabilitation, improving VO_2max_ and cardiac performance [19]. VO_2max_ improvement across exercise intensities demonstrates how tailored regimens enhance exercise tolerance in HF patients [52]. HIIT significantly improves exercise capacity and reduces symptoms, offering a practical approach to managing HF without invasive treatments [19,52]. However, exercise intolerance is a hallmark of HF. Recent studies [53] offer a comprehensive analysis of the underlying mechanisms. Del Buono and colleagues [53] analyzed its underlying mechanisms, identifying multisystem dysfunction—including impaired cardiac reserve, pulmonary issues, and skeletal muscle problems—as major contributors. This dysfunction decreases physical activity, reducing quality of life and increasing mortality. Comorbidities like diabetes, obesity, and lung diseases worsen exercise intolerance, further reducing capacity.

Cardiac Rehabilitation (CR) has been widely recognized as a key therapeutic strategy in HF management, offering numerous benefits including improved quality of life, increased exercise capacity, and reductions in hospitalizations [54]. The JACC Expert Panel showed the comprehensive nature of CR, which combines exercise training with lifestyle modifications and psychosocial support to optimize outcomes for heart failure patients [54]. Despite its well-established benefits, CR remains underutilized globally, with participation rates ranging from only 10–30 %. The Expert Panel calls for CR to be prioritized as a standard of care in heart failure management to help reduce healthcare burdens and improve patient outcomes [54].

The REHAB-HF trial [55] showed that tailored physical rehabilitation for older, frail HF patients improved strength, balance, mobility, and endurance, enhancing walking endurance and balance. However, it did not significantly impact rehospitalization rates or mortality compared to common care [55]. HIIT in particular stands out, showing significant reductions in mortality and improvements in long-term survival for heart failure patients with HF who engage in HIIT programs.

A meta-analysis of 85 randomized controlled trials involving over 23,000 participants demonstrated that exercise-based CR reduces cardiovascular mortality, myocardial infarctions, and hospitalizations in patients with coronary heart disease [21]. Also, CR was found to improve health-related quality of life and is considered cost-effective. A previous study on multidisciplinary CR in HF patients found a 23 % reduction in all-cause mortality and a 33 % reduction in HF-related rehospitalizations [56]. Including patients with varying frailty and ejection fractions, the study highlights CR's crucial role in improving survival and easing healthcare burdens by reducing hospital readmissions.

### 3.6 Innovative Exercise Interventions in heart failure

A recent pilot study [57] explored the effectiveness of HIIT supplemented with peripheral resistance training and inspiratory resistance training in patients with chronic heart failure. The study compared this combined protocol, referred to as RHIIT, with a standard exercise-based rehabilitation program. Findings demonstrated that the RHIIT program significantly improved both peripheral muscle strength and inspiratory respiratory muscle strength, with similar enhancements in VO_2peak_ as the standard program. Notably, the RHIIT protocol achieved these results in a shorter timeframe, suggesting it may offer a more efficient approach to improving physical function in patients with HF.

Traditional HIIT can be combined with peripheral resistance training (e.g., weightlifting) and inspiratory resistance training (breathing exercises) to improve VO_2peak_ and muscle strength, including in limbs and respiratory muscles, in a shorter time frame [58]. Another method, remote ischemic conditioning (RIC), involves brief periods of reduced blood flow to a limb, helping the heart build resilience against ischemic events. HF patients who underwent RIC showed improved New York Heart Association (NYHA) functional class and enhanced left ventricular ejection fraction (a crucial marker of systolic heart function) [40,50].

Tailored rehabilitation programs have shown promise in older, frail HF patients [59]. The REHAB-HF trial [55,59] evaluated a program targeting strength, balance, mobility, and endurance, leading to improvements in walking endurance and balance, though no significant changes in rehospitalization or mortality were observed. HIIT, however, has shown significant reductions in mortality and improved long-term survival in HF patients [55].

#### 3.6.1 Risks and safety considerations in long-term and high-intensity exercise

The ICFSR expert [60] consensus recommends tailored exercise for older adults, focusing on aerobic, resistance, balance, and flexibility training to reduce chronic disease risks and improve mobility. Intense workouts may pose risks for high-risk individuals, such as irregular heartbeats or rare heart damage. Regular screening is essential before starting vigorous exercise, particularly for older adults, HF patients, or those with a family history of heart disease, ensuring exercise plans are personalized based on heart health, fitness level, and conditions.

Activities like walking, cycling, or water exercises can be done at a moderate pace, while resistance bands or lightweight exercises help build muscle strength and combat sarcopenia. Yoga, Tai Chi, and stretching improve flexibility, balance, and coordination, reducing stress and fall risks in older HF patients. The integration of technology, such as a home-based CR program delivered via smartphone in China, has shown significant clinical improvements, particularly for those with coronary heart disease [61]. Patients in the intervention group experienced a lower incidence of major adverse cardiac events, improved exercise capacity, and fewer unscheduled readmissions.

Exercise responses differ significantly between heart failure subtypes, particularly HFrEF (HF with reduced ejection fraction) versus HFpEF (HF with preserved ejection fraction). Exercise limitations differ between HFrEF and HFpEF due to distinct pathophysiological mechanisms [62,63]. In patients with HF, body composition distinctly influences pulmonary oxygen uptake kinetics, skeletal muscle oxygenation, and exercise tolerance depending on the ejection fraction subtype. In HFrEF, reduced exercise capacity is mainly driven by impaired cardiac output and severely diminished stroke volume, despite near-maximal oxygen extraction by muscles. In contrast, HFpEF involves both central and peripheral limitations, including chronotropic incompetence, diastolic dysfunction, vascular stiffness, endothelial dysfunction, poor muscle perfusion, and mitochondrial inefficiency [62,64]. Compared with HFpEF, HFrEF patients exhibit significantly slower VO_2_ kinetics and faster skeletal muscle deoxygenation response, despite showing higher microvascular O_2_ delivery; exercise intolerance occurs earlier in HFrEF [62]. Even though aerobic and HIIT can improve functional capacity and quality of life in HFpEF, their effects on cardiac structure and diastolic function are generally more limited compared to HFrEF. Optimizing body composition, reducing fat and increasing lean muscle, could improve oxygen delivery and exercise tolerance, particularly in HFrEF patients [62].

**Fig. 3:**
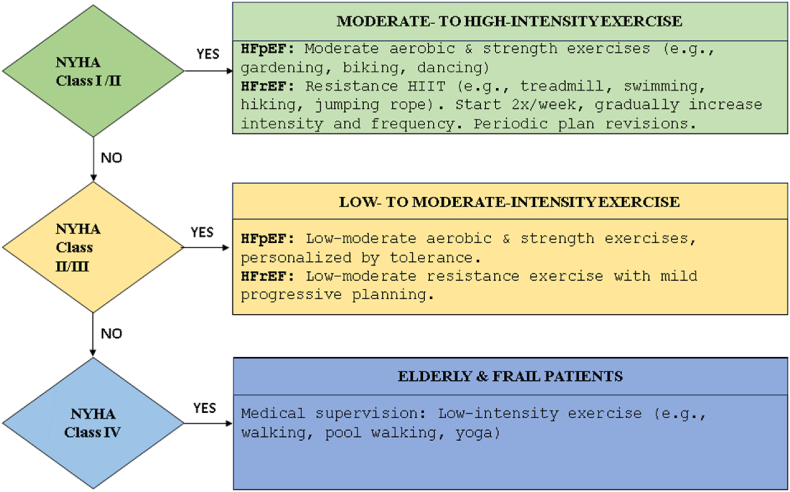
Exercise intensities for patients with various NYHA (New York Heart Association) classifications.

Elderly heart failure patients require comprehensive management, including nutritional support, pharmacotherapy, and technology-assisted home care. Exercise recommendations include low to moderate-intensity aerobic exercise (3–5 times per week), resistance training (2–3 times per week), and daily flexibility and balance exercises. Supervision and gradual progression are essential. Fig. 3 provides a flow chart detailing exercise intensities for patients with various NYHA classifications, offering further guidance on tailoring exercise programs based on heart failure severity.

Nutritional interventions, such as protein and omega-3 supplementation, along with fluid monitoring and sodium restriction, are critical. Pharmacological management with beta-blockers, ACE inhibitors, and angiotensin receptor blockers mitigates symptoms and slows disease progression. Ivabradine enhances exercise tolerance and cardiac function, particularly in older patients. Technology-assisted home care, utilizing wearables and telemedicine, facilitates continuous monitoring of cardiac function and ensures safe rehabilitation at home [65].

## 4 Discussion

### 4.1 Potential biases and patient populations

Despite the positive impact of exercise training and rehabilitation in patients with HF demonstrated by the reviewed studies, several limitations and potential biases should be considered. Many of the studies primarily involve specific patient populations, which may limit the generalizability of their findings. For example, while tailored rehabilitation programs, such as HIIT and peripheral resistance training, show promising results in improving exercise capacity and quality of life, the majority of the trials focus on relatively healthy, non-frail participants, leaving a gap in evidence for more frail or elderly HF patients who may not be able to tolerate such intensive regimens. Additionally, some studies rely on self-reported outcomes, such as quality of life measures, which may introduce reporting bias. Furthermore, many of the trials suffer from small sample sizes and short follow-up periods, limiting the ability to conclude long-term effects on mortality, hospital readmissions, and overall survival. The lack of consistency in exercise protocols and outcome measures across studies also makes it difficult to compare results and draw definitive conclusions about the most effective exercise regimens for patients with HF. Potential biases in the literature may stem from publication bias, where studies with positive results are more likely to be published, leading to an overestimation of the benefits of exercise interventions. In addition, there is a tendency for studies to focus on specific subgroups of patients, such as those with stable heart failure, while neglecting those with more complex comorbidities or severe functional impairments. Given these limitations, further research is needed to understand the long-term benefits and risks of exercise training in diverse HF populations, including frail individuals, and to develop standardized protocols that can be widely implemented in clinical practice. Research that includes larger and more diverse patient samples, longer follow-up periods, and rigorous controls for potential biases would help to provide a more balanced and comprehensive understanding of the role of exercise in HF rehabilitation.

Targeted exercise enhances mitochondrial function, substrate utilization, and endothelial health, thereby improving exercise tolerance and quality of life. Personalized exercise regimens, tailored to the individual needs of elderly HF patients, are essential for optimizing outcomes. This includes considering their comorbidities, physical capabilities, and preferences. A multidisciplinary approach involving cardiologists, physiotherapists, dietitians, and primary care providers is crucial to address the comprehensive needs of HF patients. Long-term monitoring and follow-up are necessary to ensure sustained benefits and address emerging issues promptly. Additionally, integrating physical, nutritional, and psychological support is key to enhancing the overall effectiveness of HF rehabilitation.

### 4.2 Limitations and gaps in heart failure exercise rehabilitation research

Research on exercise-based HF rehabilitation faces limitations such as small sample sizes, heterogeneous populations (age, gender, comorbidities, HF phenotypes), short follow-ups, and underrepresentation of regions like Asia, Africa, and Latin America. Limited inclusion of women, elderly, and minority groups, along with diverse exercise protocols and cultural attitudes, hinders standardization and generalizability. Randomized protocols and varied definitions of exercise intensity complicate comparisons, while long-term adherence and real-world efficacy remain underexplored. Future studies should expand geographically, include diverse populations, and explore interactions between exercise and pharmacological therapies to enhance HF rehabilitation's applicability and effectiveness.

## 5 Conclusion

This review examined the effects of exercise intensity on cardiac adaptation, focusing on myocardial bioenergetics, mitochondrial function, and heart performance in HF patients. Future research should aim to uncover the molecular mechanisms underlying these benefits, identify predictive biomarkers, and evaluate innovative approaches such as RHIIT, remote ischemic conditioning, and tech-assisted home care.

Emphasizing diverse contexts, comorbidities, emerging therapies, and digital tools will further optimize personalized care and advance HF rehabilitation outcomes. Enhancing patient education on the importance of exercise and minimizing sedentary behavior empowers patients to take an active role in their rehabilitation, ultimately leading to better management of HF.

## CRediT authorship contribution statement

**Morteza Esmaeili:** Writing – review & editing, Writing – original draft, Visualization, Methodology, Conceptualization. **Riyas Vettukattil:** Writing – review & editing, Writing – original draft, Visualization, Conceptualization.
